# Role of extracellular DNA in *Enterococcus faecalis* biofilm formation and its susceptibility to sodium hypochlorite

**DOI:** 10.1590/1678-7757-2018-0699

**Published:** 2019-07-25

**Authors:** Mi-Kyung YU, Mi-Ah KIM, Vinicius ROSA, Yun-Chan HWANG, Massimo DEL FABBRO, Won-Jun SOHN, Kyung-San MIN

**Affiliations:** 1 Chonbuk National University Chonbuk National University School of Dentistry and Institute of Oral Bioscience Department of Conservative Dentistry Jeonju Korea Chonbuk National University, School of Dentistry and Institute of Oral Bioscience, Department of Conservative Dentistry, Jeonju, Korea.; 2 Chonbuk National University Chonbuk National University Research Institute of Clinical Medicine Jeonju Korea Chonbuk National University, Research Institute of Clinical Medicine, Jeonju, Korea.; 3 Chonbuk National University Hospital Chonbuk National University Hospital Biomedical Research Institute Jeonju Korea Chonbuk National University Hospital, Biomedical Research Institute, Jeonju, Korea.; 4 National University of Singapore National University of Singapore Faculty of Dentistry Singapore National University of Singapore, Faculty of Dentistry, Discipline of Oral Sciences, Singapore.; 5 Chonnam National University Chonnam National University School of Dentistry Department of Conservative Dentistry Gwangju Korea Chonnam National University, School of Dentistry, Department of Conservative Dentistry, Gwangju, Korea.; 6 Università degli Studi di Milano Università degli Studi di Milano Dipartimento di scienze biomediche, chirurgiche e odontoiatriche Milan Italy Università degli Studi di Milano, Dipartimento di scienze biomediche, chirurgiche e odontoiatriche, Milan, Italy.; 7 IRCCS Istituto Ortopedico Galeazzi IRCCS Istituto Ortopedico Galeazzi Milan Italy IRCCS Istituto Ortopedico Galeazzi, Milan, Italy; 8 Seoul National University Seoul National University Dental Research Institute and School of Dentistry Department of Conservative Dentistry Seoul Korea Seoul National University, Dental Research Institute and School of Dentistry, Department of Conservative Dentistry, Seoul, Korea.

**Keywords:** DNA, Enterococcus faecalis, Sodium hypochlorite, Biofilms

## Abstract

**Objective:**

This study investigated the role of extracellular deoxyribonucleic acid (eDNA) on *Enterococcus faecalis* ( *E. faecalis* ) biofilm and the susceptibility of *E. faecalis* to sodium hypochlorite (NaOCl).

**Methodology:**

*E. faecalis* biofilm was formed in bovine tooth specimens and the biofilm was cultured with or without deoxyribonuclease (DNase), an inhibitor of eDNA. Then, the role of eDNA in *E. faecalis* growth and biofilm formation was investigated using colony forming unit (CFUs) counting, eDNA level assay, crystal violet staining, confocal laser scanning microscopy, and scanning electron microscopy. The susceptibility of *E. faecalis* biofilm to low (0.5%) or high (5%) NaOCl concentrations was also analyzed by CFU counting.

**Results:**

CFUs and biofilm formation decreased significantly with DNase treatment (p<0.05). The microstructure of DNase-treated biofilms exhibited less structured features when compared to the control. The volume of exopolysaccharides in the DNase-treated biofilm was significantly lower than that of control (p<0.05). Moreover, the CFUs, eDNA level, biofilm formation, and exopolysaccharides volume were lower when the biofilm was treated with DNase de novo when compared to when DNase was applied to matured biofilm (p<0.05). *E. faecalis* in the biofilm was more susceptible to NaOCl when it was cultured with DNase (p<0.05). Furthermore, 0.5% NaOCl combined with DNase treatment was as efficient as 5% NaOCl alone regarding susceptibility (p>0.05).

**Conclusions:**

Inhibition of eDNA leads to decrease of *E. faecalis* biofilm formation and increase of susceptibility of *E. faecalis* to NaOCl even at low concentrations. Therefore, our results suggest that inhibition of eDNA would be beneficial in facilitating the efficacy of NaOCl and reducing its concentration.

## Introduction

*Enterococcus faecalis* ( *E. faecalis* ) is a bacterium frequently recovered from infected root canal systems.^1 , 2^ This bacteria is difficult to remove since it is able to form biofilms and survive under a wide range of acidic and basic conditions and prolonged periods of nutritional deprivation.^3 , 4^ *E. faecalis* biofilms consist of exopolysaccharides, proteins, lipids, and extracellular deoxyribonucleic acid (eDNA).^5 , 6^ The dense and protected environment of a biofilm may facilitate gene transfer and enhance biofilm stability.^7^ As a major structural component of many different microbial biofilms, the importance of eDNA was first reported in *Pseudomonas aeruginosa* .^5^ The eDNA is released via autolysis in a fratricidal or suicidal manner and/or active release through membrane vesicles and nanofibers in *E. faecalis* biofilms.^6 , 8^ Previous reports have attributed a crucial role to eDNA in the formation, mechanical stability, and maturation of bacterial biofilms in general and *E. faecalis* biofilms in particular.^9 - 11^ Irrigation is critical to remove microorganisms from root canal systems. NaOCl is the most frequently used material for endodontic treatment, it is an antiseptic and inexpensive lubricant that has been used at dilutions ranging from 0.5% to 5.25%.^12^ Usually, it is assumed that a higher concentration of NaOCl increases the efficacy in removing bacteria within root canal systems; however, severe complications of NaOCl extrusion during endodontic treatment can occur at high concentrations.^13^ NaOCl is a strong oxidizing agent and may cause significant damage when in direct contact with tissue, including rapid hemolysis and ulceration, inhibition of neutrophil migration, and destruction of endothelial and fibroblast cells.^14^ Therefore, there have been several alternative approaches proposed to improve the effectiveness of lower-concentration NaOCl solutions to avoid extensive tissue damage; however, these options have many limitations, including the requirement of inconvenient equipment.^15 - 19^ Since eDNA is an essential component of *E. faecalis* biofilm, it can be speculated that its inhibition using a simple agent can be another strategy for effective biofilm removal. However, to our knowledge, there has been little information regarding the effect of eDNA on *E. faecalis* biofilm in endodontic study models. Therefore, the aim of this study was to investigate the role of eDNA in the formation of *E. faecalis* biofilm in bovine root canal systems through DNase, a known eDNA inhibitor. Moreover, this study highlights the susceptibility of the bacteria to NaOCl by eliminating eDNA in the biofilm.

## Methodology

### Preparation of specimens

**Figure 1:**
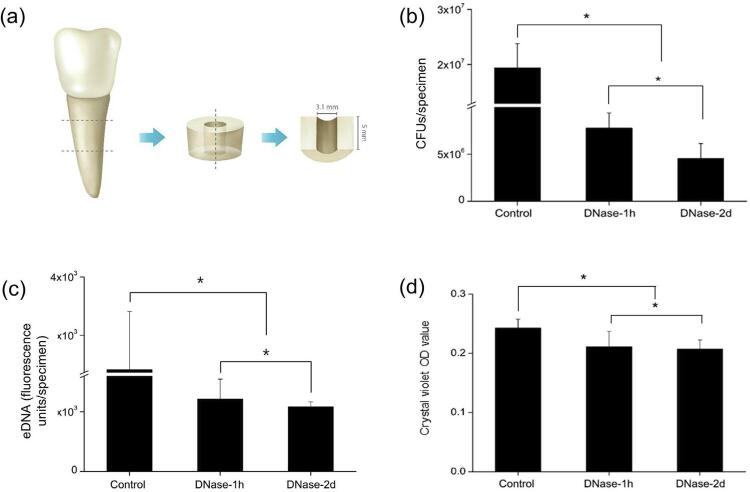
(a) Schematic illustration of the bovine root canal specimen. (b) CFUs of *E. faecalis* biofilms from the specimens. (c) eDNA measured using SYBR green. (d) Biofilm formation measured by crystal violet staining. Control: cultured only in BHI for 2 days, DNase-1h: treated with DNase for 1 hour after 2-day culture in BHI, and DNase-2d: cultured with DNase for 2 days. *Statistical significance was determined at p<0.05

This study used recently extracted single-rooted bovine central incisors. The teeth were immersed in 1% NaOCl solution for 24 h for surface disinfection. Following, each tooth was horizontally sectioned 5 mm in length below the cementoenamel junction using a diamond saw (AEU-25, Aseptico, Woodinville, WA, USA) at 15,000 rpm. The root canals of the cylindrical specimens were enlarged using a 3.1 mm diameter round bur. Next, each specimen was vertically sectioned with a diamond saw into cylindrical halves ( Figure 1a ). Finally, the smear layer was removed using 17% ethylenediaminetetraacetic acid solution. To prevent bacterial contamination, the specimen was steam-sterilized in an autoclave (LAC-5101SD, DAIHAN Lab Tech, Namyangju, Korea) at 121°C for 20 minutes. To ensure dentin contamination only through the main root canal wall, the outer surface of the sectioned specimens was varnished with a double layer of nail polish.

### Infection of the specimens and DNase treatment

*E. faecalis* (bacterial strain ATCC 29212) was aerobically cultured in sterile brain heart infusion (BHI; Difco Laboratories, Detroit, MI, USA) medium at 37°C. The BHI plates contained 1.5% (wt/vol) of agar (Difco Laboratories). The specimens were placed vertically in a 48-well plate (SPL, Daejeon, Korea), and *E. faecalis* (6×10^5^CFU/mL) was transferred to each well. Following, the plates were incubated at 37°C for 2 days and divided into 3 groups (n=9): (i) *E. faecalis* in BHI (control), (ii) *E. faecalis* in BHI with DNase (ELPIS, Daejeon, Korea) treatment at 2 U/μl/ml for 1 h after 2-day incubation (DNase-1h), and (iii) *E. faecalis* and DNase (2 U/μl/ml) in BHI (DNase-2d).

### Colony forming unit (CFU) counting and eDNA measurement

The specimens were washed with sterile water and then transferred into a 1.5 ml tube containing 1 ml of sterile water. Following, the specimens were sonicated to collect eDNA using a sonifier (10 s, two times at 20% energy level) (VCX 130PB; Sonics & Materials, Newtown, CT, USA). CFU counting was performed by plating serial dilution of an aliquot (0.1 ml) of each specimen on BHI agar plates. The rest were centrifuged at 10000× *g* at 4°C for 10 min and the supernatant was filtered. For eDNA measurement, the supernatant was treated with DNA-binding dye, SYBR Green (Invitrogen, Carlsbad, CA, USA). eDNA concentration was measured with excitation at 485 nm and emission at 535 nm using a fluorescence microplate reader (HIDEX, Turku, Finland).

### Crystal violet staining

Crystal violet staining was performed to assess the biofilm mass in the specimens. The specimens were washed with sterile water and added 0.1% crystal violet (Sigma-Aldrich, St. Louis, MO, USA). Following, the specimens were kept at room temperature for 10 min. Next, they were rinsed with sterile water, treated with 30% acetic acid (Fisher Scientific, Fair Lawn, NJ, USA), and transferred to a 96-well plate. The absorbance at 595 nm was then measured (µQuant, Biotek Instrument, Winooski, VT, USA).

### Confocal laser scanning microscopy (CLSM)

One μM of Alexa Fluor 647-labeled dextran conjugate (Molecular Probes, Eugene, OR, USA) was added to the BHI broth with *E. faecalis* (6×10^5^ CFU/ml), and then incubated for 2 days at 37°C. The fluorescence-labeled dextran was incorporated during exopolysaccharide matrix synthesis over the course of biofilm development. After 2 days, 2.5 μM SYTO 9 green fluorescent nucleic acid stain (480/500 nm; Molecular Probes) was added and incubated for 30 min to label the bacteria in the biofilms. Following, CLSM imaging was conducted using an LSM 510 META microscope (Carl Zeiss, Jena, Germany) (EC Plan-Neofluar 10×/0.30 M27). Three independent experiments were performed and seven image stacks (512×512 pixel tagged image file format) were collected per experiment. For the measurement of exopolysaccharides thickness, 512×512 pixel fields were collected in a stack of seven slices at a 7.86 µm interval. The exopolysaccharides volumes were quantified from the confocal stacks using COMSTAT (www.comstat.dk; Kongens Lyngby, Denmark).^20^

### Scanning electron microscopy (SEM)

The biofilm formed on the specimens was rinsed with phosphate buffered saline (Sigma-Aldrich) and fixed in 2.5% glutaraldehyde (Sigma-Aldrich) for 2 h at 4°C. Following, the specimens were dehydrated in a graded series of ethanol (25–100%) and air-dried them at room temperature for 1 h. The samples were coated with gold-palladium and observed using SEM (Hitachi, Tokyo, Japan). The images were obtained on a Hitachi SU-70 using BSE detector with 10.0 Kv voltage acceleration.

### NaOCl treatment and CFU counting

To study the *E. faecalis* susceptibility to NaOCl in biofilm treated with DNase, the specimens with *E. faecalis* (6×10^5^ CFU/ml) and DNase (2 U/μl/ml) were cultured for 2 days at 37°C. After incubation, the biofilms formed on the specimens were treated with 0.5% or 5% NaOCl for 5 min. Next, the specimen was transferred to a 1.5 ml tube that contained 1 ml of sterile water and sonicated to break the biofilms. CFU counting was performed by plating serial dilution of an aliquot (0.1 ml) of each specimen on BHI agar plates.

### Statistical analysis

All experiments were performed at least three times. Data was analyzed using 1-way analysis of variance, followed by Tukey’s test. The data were presented as mean and standard deviation. Statistical significance was considered when *p* >0.05. These analyses were performed with the SPSS software (SPSS 12.0 K for Windows; SPSS Inc., Chicago, IL, USA).Discussion

eDNA is an important component in the extracellular matrix of *E. faecalis* biofilms.^5 , 6^ Researchers have investigated the function of eDNA to control biofilm formation since bacterial biofilms are related to persistent infections and provide strong bacterial resistance against antimicrobial agents.^21 - 25^ *E. faecalis* is commonly isolated from failed root canal systems due to its ability to survive conventional endodontic treatment and periods of nutrient limitation and other challenging growth conditions.^3 , 26^ The sensitivity of *E. faecalis* biofilms – particularly early in development – to DNase has been previously reported.^5 , 27^ We hypothesized that a clinical model of EF biofilm formation would show decreased biomass by combining eDNA reduction and NaOCl treatment.

First, we investigated the effects of eDNA inhibition on the growth of *E. faecalis per se* and its biofilm formation. With DNase, the inhibitor of eDNA used in this clinical model, it was important to determine the duration of its application into the infected root canal system. For this end, we cultured *E. faecalis* with DNase for 2 days or treated the biofilms that had formed for 2 days with DNase for 1 hour. The results showed that *E. faecalis* and its biofilm, as well as eDNA, were removed more effectively when cultured in the presence of DNase when compared to treatment with DNase after biofilm formation ( Figure 1b-d ). A recent study reported that DNase treatment does not disperse 24-hour matured *E. faecalis* biofilms formed in root canal systems.^28^ Furthermore, some studies have demonstrated that eDNA played an important role in the early stage of biofilm formation.^6 , 10^ Our results are consistent with a model in which early exposure of DNase to the biofilm may be more effective for *E. faecalis* biofilm formation inhibition when compared to the short-term treatment of DNase to matured biofilm.

**Figure 2:**
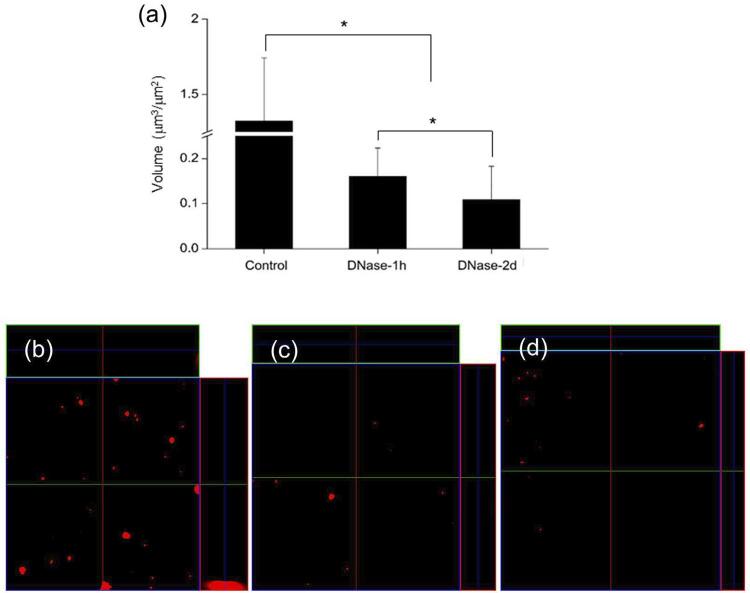
(a) Exopolysaccharides volume in the biofilms. (b-d) Representative CLSM images for measuring exopolysaccharides. Control: cultured only in BHI for 2 days, DNase-1h: treated with DNase for 1 hour after 2-day culture in BHI, and DNase-2d: cultured with DNase for 2 days. *Statistical significance was determined at p<0.05

**Figure 3:**
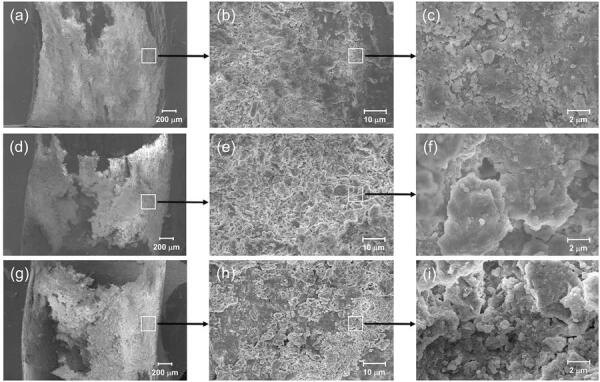
SEM images of *E. faecalis* biofilm formed in the bovine root canal specimens. (a-c): cultured only in BHI for 2 days (Control), (d-f): treated with DNase for 1 hour after 2-day culture in BHI (DNase-1h), and (g-i): cultured with DNase for 2 days (DNase-2d). Magnification: (a, d, and g) - 30X, (b, e, and h) - 1000X, and (c, f, and i) - 50000X

Next, we identified the structure of the *E. faecalis* biofilm by CLSM and SEM according to DNase treatment. We detected exopolysaccharides surrounding bacterial cells *in situ* using CLSM to further confirm the potential of biofilm formation in *E. faecalis* . The results showed that the DNase-treated groups exhibited a significantly lower exopolysaccharides volume when compared to the control group ( Figure 2a-d ). Moreover, the exopolysaccharides volume of the DNase-2d group was significantly lower than that of the DNase-1h group. In fact, polysaccharides are believed to be a major prerequisite for biofilm formation.^29^ Notably, there have been several studies showing the existence of a correlation between eDNA and exopolysaccharides.^30 - 32^ In this regard, the dimensional evaluation of exopolysaccharides may reflect the amount and integrity of the biofilm. Furthermore, this evaluation can be an alternative method for the crystal violet staining. The staining quantification is known to be less useful in determining clinically-relevant biofilm factors, although being0 generally used to evaluate biofilm biomass *in toto* .^33^ In SEM observation, the microstructure of DNase-treated biofilms exhibited more disintegrated characteristics when compared to the control ( Figure 3 ). Previous studies demonstrated that eDNA was required to stabilize the biofilm structure, and the addition of exogenous DNases could prove a potent strategy for controlling biofilm growth.^7 , 34^ Therefore, it can be suggested that DNase might hamper biofilm formation by producing structure defects in the extracellular matrix. Corroborating previous studies, our results obtained by CLSM and SEM indicate that eDNA removal may interfere with the structural integrity of the nascent biofilm.

Lastly, we investigated the effect of eDNA removal from the biofilm on the susceptibility of *E. faecalis* to NaOCl. NaOCl is the most widely used material to irrigate in dental procedures due to its high antimicrobial activity and capacity to dissolve organic tissue.^35^ However, NaOCl has drawbacks including an unpleasant odor and high toxicity that induces irritation when in contact with surrounding tissue.^36^ Therefore, in an attempt to reduce the NaOCl concentration, we used DNase and investigated whether DNase increases the efficacy of NaOCl against *E. faecalis* in biofilms. According to the results of this study, 5% NaOCl exhibited a higher antibacterial effect against *E. faecalis* than 0.5% with or without DNase. However, the gap in elimination efficiency between 5% and 0.5% NaOCl became smaller when DNase was used, mainly for potentiation of the effect of the low NaOCl concentration. Notably, 0.5% NaOCl combined with DNase exhibited statistically similar values to 5% NaOCl without DNase, suggesting that DNase adjunctive treatment could represent a useful strategy for improving antimicrobial action while reducing NaOCl concentration.

## Results

### Effect of eDNA on *E. faecalis* biofilm formation and stability

The DNase-treated groups exhibited significantly fewer CFUs when compared to the control ( *p* <0.05) ( Figure 1b ). Furthermore, the DNase-2d group showed fewer CFUs when compared to the DNase-1h group ( *p* <0.05). eDNA levels collected from the specimens of each group showed that there was also a significant decrease in DNase treated groups ( *p* <0.05) ( Figure 1c ). Crystal violet staining showed significantly decreased biofilm formation by 13% (DNase-1h) and 15% (DNase-2d) when compared to the control ( *p* <0.05) ( Figure 1d ). Similar to the CFU counting results, there was a significant difference between the two experimental groups ( *p* <0.05). As shown in Figure 2a-d , CLSM analysis showed that the exopolysaccharides volume in the DNase-treated biofilm was significantly lower than the control ( *p* <0.05). Moreover, there was a statistical difference between the DNase-1h and DNase-2d groups in terms of exopolysaccharides volume ( *p* <0.05). In SEM observation, DNase-treated biofilms exhibited more porous and disintegrated characteristics when compared to the untreated control ( Figure 3 ).

### Effect of DNase on the susceptibility of *E. faecalis* in the biofilm to NaOCl

**Figure 4:**
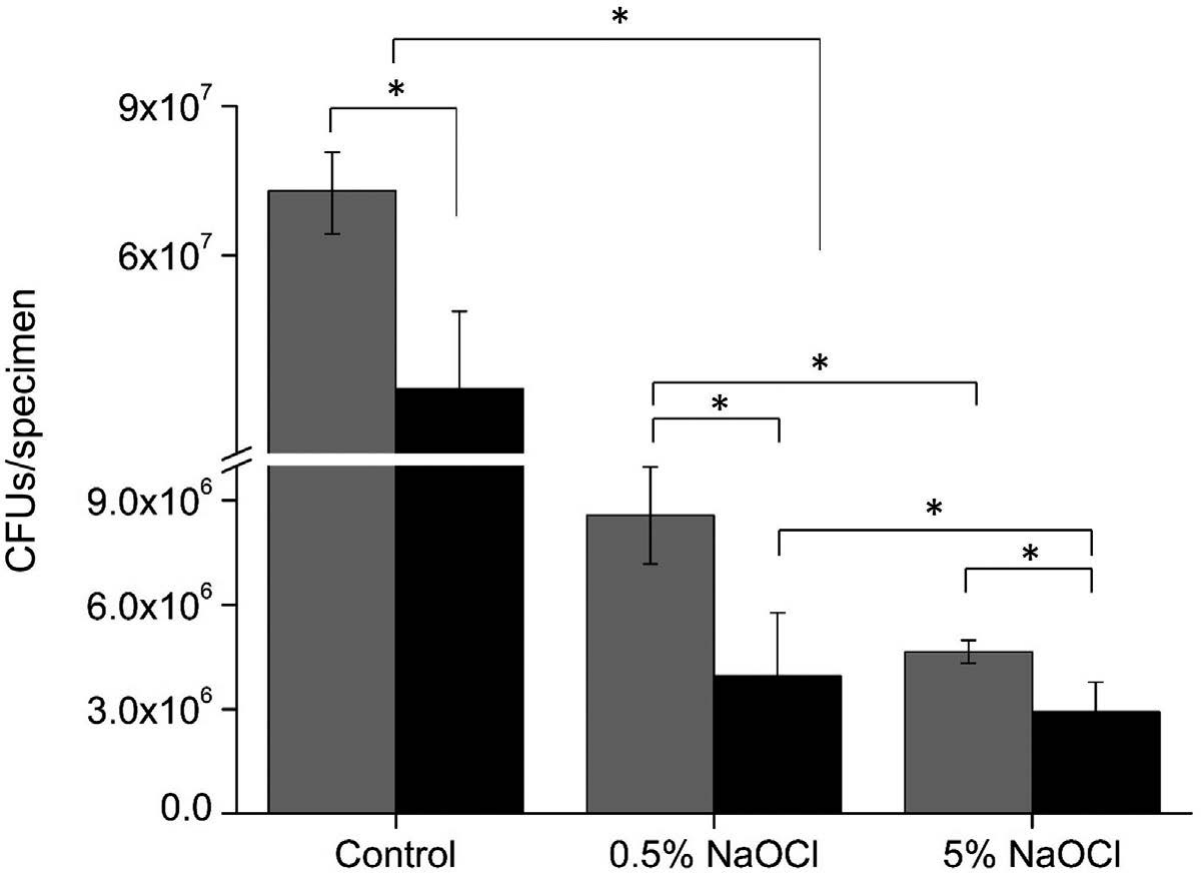
CFU assay with NaOCl treatment. *E. faecalis* were cultured in BHI without DNase (gray bar) or with DNase (black bar) for 2 days, and then the biofilms were treated with 0.5 or 5% of NaOCl for 5 minutes. Control: cultured only in BHI for 2 days without NaOCl treatment. *Statistical significance was determined at p<0.05

To investigate whether removal of eDNA from biofilms enhanced the susceptibility of *E. faecalis* to NaOCl within the biofilms, 0.5% and 5% NaOCl were used to treat the biofilms with or without DNase for 2 days. As shown in Figure 4 , the biofilms treated with NaOCl had significantly fewer CFUs when compared to the control that received no NaOCl treatment ( *p* <0.05). Regardless of the concentration, NaOCl had significantly higher efficiency in eliminating *E. faecalis* when it was used with DNase ( *p* <0.05). Furthermore, 5% NaOCl exhibited a higher bactericidal effect against *E. faecalis* than 0.5% with or without DNase. However, 0.5% NaOCl with DNase showed similar efficacy to 5% NaOCl without DNase ( *p* >0.05).

**

## Conclusions

The results suggest that inhibition of eDNA leads to decrease of *E. faecalis* biofilm formation and increase *E. faecalis* susceptibility to NaOCl especially at low concentration (0.5%). Therefore, our results suggest that inhibition of eDNA would be beneficial for removal of *E. faecalis* biofilm, especially by facilitating the efficacy of NaOCl and reducing its concentration for safer clinical use.
